# Treatments for Female Victims of Intimate Partner Violence: Systematic Review and Meta-Analysis

**DOI:** 10.3389/fpsyg.2022.793021

**Published:** 2022-02-04

**Authors:** Günnur Karakurt, Esin Koç, Pranaya Katta, Nicole Jones, Shari D. Bolen

**Affiliations:** ^1^Department of Psychiatry, Case Western Reserve University, Cleveland, OH, United States; ^2^Department of Psychology, Başkent University, Ankara, Turkey; ^3^Department of Psychiatry, University Hospitals Cleveland Medical Center, Cleveland, OH, United States; ^4^Population Health Research Institute, MetroHealth Medical Center, Cleveland, OH, United States; ^5^Center for Health Care Research and Policy, Case Western Reserve University at the MetroHealth Medical Center, Cleveland, OH, United States; ^6^Department of Medicine, MetroHealth Medical Center, Cleveland, OH, United States; ^7^Department of Population and Quantitative Health Sciences, Case Western Reserve University, Cleveland, OH, United States

**Keywords:** intimate partner violence, systematic review, meta-analysis, domestic violence, treatment, women's health

## Abstract

Intimate partner violence (IPV) is an important problem that has significant detrimental effects on the wellbeing of female victims. The chronic physical and psychological effects of intimate partner violence (IPV) are complex, long-lasting, chronic, and require treatments focusing on improving mental health issues, safety, and support. Various psycho-social intervention programs are being implemented to improve survivor wellbeing. However, little is known about the effectiveness of different treatments on IPV survivors' wellbeing. For this purpose, we conducted a systematic review and meta-analysis to assess the effectiveness of interventions on improving outcomes that describe the wellbeing of adult female survivors of IPV. We searched PubMed, PsycINFO, and Cochrane Library. We explored the effectiveness of available interventions on multiple outcomes that are critical for the wellbeing of adult female victims of IPV. To provide a broad and comprehensive view of survivors' wellbeing, we considered outcomes including mental health, physical health, diminishing further violence, social support, safety, self-efficacy, and quality of life. We reviewed 2,770 citations. Among these 25 randomized-controlled-study with a total of 4,683 participants met inclusion criteria. Findings of meta-analyses on interventions indicated promising results in improving *anxiety* [standardized mean difference (SMD) −7.15, 95% confidence interval (CI) −8.39 to −5.92], *depression* (SMD −0.26, CI −0.56 to −0.05), *safety* (SMD = 0.43, CI 0.4 to −0.83), *violence prevention* (SMD = −0.92, CI −1.66 to −0.17), *health* (SMD = 0.39, CI 0.12 to 0.66), *self-esteem* (SMD = 1.33, CI −0.73 to 3.39), *social support* (SMD =0.40, CI 0.20 to 0.61), and *stress management* (SMD = −8.94, CI −10.48 to −7.40) at the post-test. We found that empowerment plays a vital role, especially when treating *depression* and *Post-Traumatic Stress Disorder (PTSD)*, which are difficult to improve across interventions. We found mixed findings on self-efficacy and quality of life. The effects of IPV are long-lasting and require treatments targeting co-morbid issues including improving safety and mental health issues.

## Effectiveness of Treatments for Adverse Effects of Intimate Partner Violence

Intimate Partner Violence (IPV) is a public health problem. It entails aggressive and violent, physical, sexual, verbal, and psychological acts by an intimate partner (Breiding et al., 2014; CDC, 2019). One in four women has reported experiencing partner violence at some point in their lifetime (Breiding et al., 2014; CDC, 2019). Over a third of women (36.4%) experience psychological aggression and about 41% of female survivors experience some form of physical injury during their lifetime (NISVS, 2012). Acute injuries including bruises, fractures, sensory damage, and internal injury, in addition to long term ailments such as muscle-skeletal issues and metabolic issues, are significantly more common among the victims of IPV than non-victims (Krug et al., 2002; Kilpatrick et al., 2003; Karakurt et al., 2017; Liu et al., 2020).

Mental health problems are also highly prevalent among the victims of IPV (Karakurt et al., 2014; Oram et al., 2017). Victims frequently report experiencing a broad range of Post-Traumatic Stress Disorder symptoms (Schnurr and Green, 2004; Afifi et al., 2009; World Health Organization, 2013; Akyazi et al., 2018). Past research also reports the association between IPV and an increased likelihood of clinical depression and suicide attempts (Mapayi et al., 2012; Akyazi et al., 2018). Comorbidities of multiple mental health issues are also common such as depression, anxiety, and PTSD among IPV victims (Schnurr and Green, 2004; World Health Organization, 2013). IPV also affects victims economically due to challenges in finding employment lost productive days, and difficulty in accessing the available resources adding to their stressors (Ford-Gilboe et al., 2009).

Researchers and clinicians developed numerous treatment programs to improve the wellbeing of victims. Initially, local shelters were provided these programs to prevent further violence and improve safety (Berk et al., 1986). Researchers found these shelter-based interventions to be beneficial for the victims in improving their current situation (Clevenger and Roe-Sepowitz, 2009). In addition, advocacy services in shelters play an important role in victims' life satisfaction by helping them to navigate community resources (Sullivan and Bybee, 1999).

Treatment programs vary in their goals, structures, and main approach as well as delivery methods such as the domestic violence shelters, community mental health agencies, and hospitals. These programs draw from a multitude of therapy models such as cognitive-behavioral therapy (CBT), mindfulness, motivational interviewing, and expressive writing. Treatment modalities based on the CBT approach focuses on changing cognitive distortions in addressing the potential issues (Butler et al., 2006), motivational approaches focus on building intrinsic motivation to change behaviors (Rollnick and Miller, 1995), stress management approaches focus on improving coping skills to deal with stressors (Nam et al., 2020) and mindfulness-based approaches focus on improved awareness to reduce depressive and anxiety symptoms (Evans et al., 2008; Piet and Hougaard, 2011).

Additionally, patients who discuss, share, and write their traumatic experiences and intrusive memories are found to have better mental health outcomes and a greater reduction in PTSD, perceived stress, and depressive symptoms (Brewin et al., 2010). Furthermore, victims receiving psychoeducational materials and advocacy interventions report higher scores with their mood and behavior, lower depressive symptoms, and more social support (Tiwari et al., 2010).

The psychosocial wellbeing of survivors is multi-faceted, and many outcomes including mental and physical health, social support, and self-efficacy are critical to the wellbeing of a survivor. However, although many different interventions exist to treat survivors of IPV, less is known about the effectiveness of these interventions. Prior systematic reviews and meta-analysis focused on the screening of IPV (Nelson et al., 2012; O'Doherty et al., 2014) or the effects of IPV on specific populations, such as pregnant women (Hill et al., 2016) and women who suffer from substance abuse (Devries et al., 2014). These studies do not specifically explore multiple critical outcomes on the psycho-social wellbeing of the victims of IPV. For this purpose, we performed a systematic review and meta-analysis to investigate the effectiveness of interventions designed to improve the psycho-social wellbeing of survivor victims and their related comorbidities.

## Methods

### Search Strategy and Identification of Studies

We followed The Cochrane Handbook for Systematic Reviews of Interventions guidelines for the process of conducting high-quality systematic reviews and meta-analyses (Higgins and Green, 2011). We searched Pubmed, PsycINFO, and Cochrane Library to access the studies that were published on the topic. We used the keywords: “battered women,” “abused women,” “victim,” “survivor,” “domestic violence,” “intimate partner violence,” “partner abuse,” “partner violence,” “spousal abuse,” “violence against women,” “battering,” and “physical abuse,” in combination with keywords “treatment,” “intervention,” “therapy,” “counseling,” “education,” “prevention,” “outcome,” and “curriculum.” Two reviewers trained and independently assessed each title, abstract, and article for eligibility.

We used the predefined Population, Intervention, Comparison, Outcomes, Time, and Setting (PICOTS) for inclusion and exclusion criteria to assess the eligibility of the studies (Higgins et al., 2019). Reviewers received training in the methodology. Studies included if (i) their sample composed of adult female victims who are suffering from physical intimate partner violence (P), (ii) they have active interventions designed for victims of IPV (I) (iii) they have a control group (C), they measure outcomes related to psycho-social wellbeing of physically abused women (O), they measure outcomes at pre-and post-intervention (T), and finally, all setting were included (S).

The exclusion criteria for the title and abstract review were the following: “no original data,” “interventions that do not contain control,” “not a peer-reviewed study,” and “women under 18 years of age.” In the article review, we also excluded studies that presented the data in such a way that did not allow the pooling of the results. In addition, we excluded studies that did not directly assess the effectiveness of the intervention for female victims of IPV on outcomes related to wellbeing. We only included studies that used true control groups, i.e., we did not abstract data from studies that utilized non-completers as the control group. We included studies with minimal controls such as a no-treatment group, safety advising pamphlets, information about community resources, referral cards, waitlist, and watching popular TV programs as the comparison group.

### Data Abstraction

Two team members abstracted data from included articles. We abstracted data serially by one researcher and then double-checked by the other researcher for accuracy. We used standard data abstraction forms to extract data on the population, study design, details about the intervention, and outcomes. The extracted data included the type of treatment, number of sessions, control, and setting. We abstracted mean and standard deviation values among intervention and control groups. Discrepancies between reviewers were resolved during the team meeting.

### Data Analysis

We combined studies when at least three studies measured the same outcome. We standardized the effect sizes. We used standardized mean difference (SMD) to pool results across studies for the same outcome since many studies used different measures. We used the *I*^2^ measure to assess heterogeneity. We conducted subgroup analyses when *I*^2^ > 50% and ten or more studies (*N* > 10) to evaluate differences by specific a priori subgroups.

### Quality of Studies

We used the Cochrane Risk of Bias Tool to evaluate the risk of bias for the included studies (Armijo-Olivo et al., 2012). These risks include selection bias, publication bias, reporting bias, performance bias, detection bias, and attrition bias. Selection bias refers to the lack of comparable groups for both intervention and control. We looked for strategies such as randomization in our assessment of the studies. Selective outcome reporting indicates reporting bias. We looked for whether studies reported their outcome of interest and related outcomes for the study and whether unfavorable outcomes were reported in the article. For performance and detection bias, we assessed whether there was blinding and whether interventions were carried out in similar conditions both for intervention and control groups. Attrition bias is present when there is incomplete outcome data. We used the drop-out rate as an indicator of attrition bias. Team members independently assessed study quality and then resolved conflicts together. If any conflicts could not be resolved, they were brought to the larger study team for resolution.

## Results

### Identification of Studies

**Figure 1:**
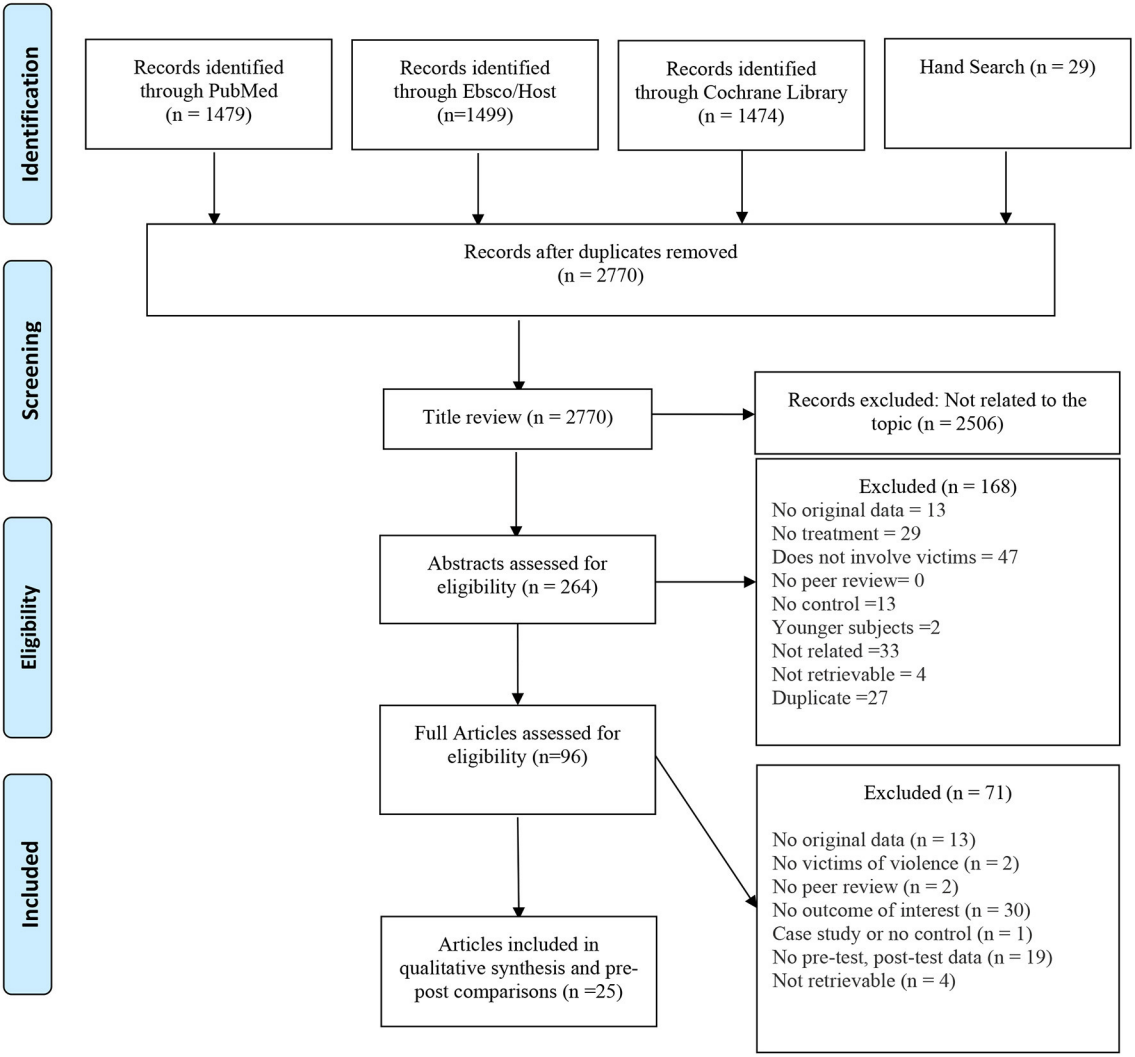
Prisma flow diagram for included studies of systematic review and meta-analysis for treatments for female victims of intimate partner violence.

Electronic databases [PubMed (*N* = 1,479), Ebsco/Host (*N* = 1,499), Cochrane Library (*N* = 1,474)] in addition to Hand Searched articles (*N* = 29) revealed a total of 2,770 articles after duplicates were removed. During the title review, 2,508 articles were excluded since they were not specifically relevant to answering the question of interest. In the title review, we screened for adult female victims who were suffering from physical IPV. Of the remaining 264 articles that underwent abstract review, 168 articles were excluded. The most frequent reasons for the exclusion of articles at this stage were that they did not include the study population (*n* = 47), did not relate to the topic (*n* = 33), or did not have treatment (*n* = 29). Of the 96 articles that underwent full article review, 70 were excluded. The most frequent reasons for the exclusion of articles at this stage were having no outcome of interest (*n* = 30), no pre- and post-test data that we could pool with the rest of the studies (*n* = 19), and no original data (*n* = 13). Twenty-five studies remained that met all the inclusion and exclusion criteria. The Prisma flow diagram for the included studies is demonstrated in Figure 1.

### Characteristics of Studies

We included a diverse set of studies in terms of the interventions they used, related co-morbidities, and psycho-social outcomes. Supplementary Table 1 shows the characteristics of these studies. Researchers conducted the studies in the United States (*n* = 14) (McFarlane et al., 2002; Kubany et al., 2004; Constantino et al., 2005; Koopman et al., 2005; Resick et al., 2008; Johnson et al., 2011, 2016; McWhirter, 2011; Zlotnick et al., 2011, 2019; Saftlas et al., 2014; Eden et al., 2015; Rhodes et al., 2015; Stevens et al., 2015; Glass et al., 2017), as well as in different countries, such as Australia (*n* = 1) (Taft et al., 2011), China (*n* = 1) (Tiwari et al., 2012), Greece (*n* = 1) (Kokka et al., 2019), India (*n* = 1) (Patel et al., 2019), Iran (*n* = 2) (Ghahari et al., 2016; Orang et al., 2018), Mexico (*n* = 1) (Gupta et al., 2017), Spain (*n* = 1) (Tirado-Muñoz et al., 2015), Turkey (*n* = 1) (Bahadir-Yilmaz and Öz, 2018), and the United Kingdom (*n* = 1) (Ferrari et al., 2018). Many of the studies had samples with high percentages of Caucasian women (38%) while 12% of the studies had a majority of African-American women in their sample and 8% of the studies had a majority of Hispanic women in their sample. In the majority of studies women aged between 30 and 50 years old.

Many of the studies conducted individual interventions with a wide range of intervention types. These interventions included cognitive-behavioral therapy (CBT, or augmented with CBT techniques), motivational interviewing, narrative expressive writing, trauma work, advocacy services, telephone social support, and safety planning aid. Different intervention studies used facilitators with different skill levels. For example, a majority of the studies included masters or Ph.D. educated therapists, while some had advocacy services provided by trained nurses and advocates. Some other studies involved an internet-based intervention that does not have a facilitator but rather has a computer interface.

### Quality of the Studies and Risk of Bias

**Figure 2:**
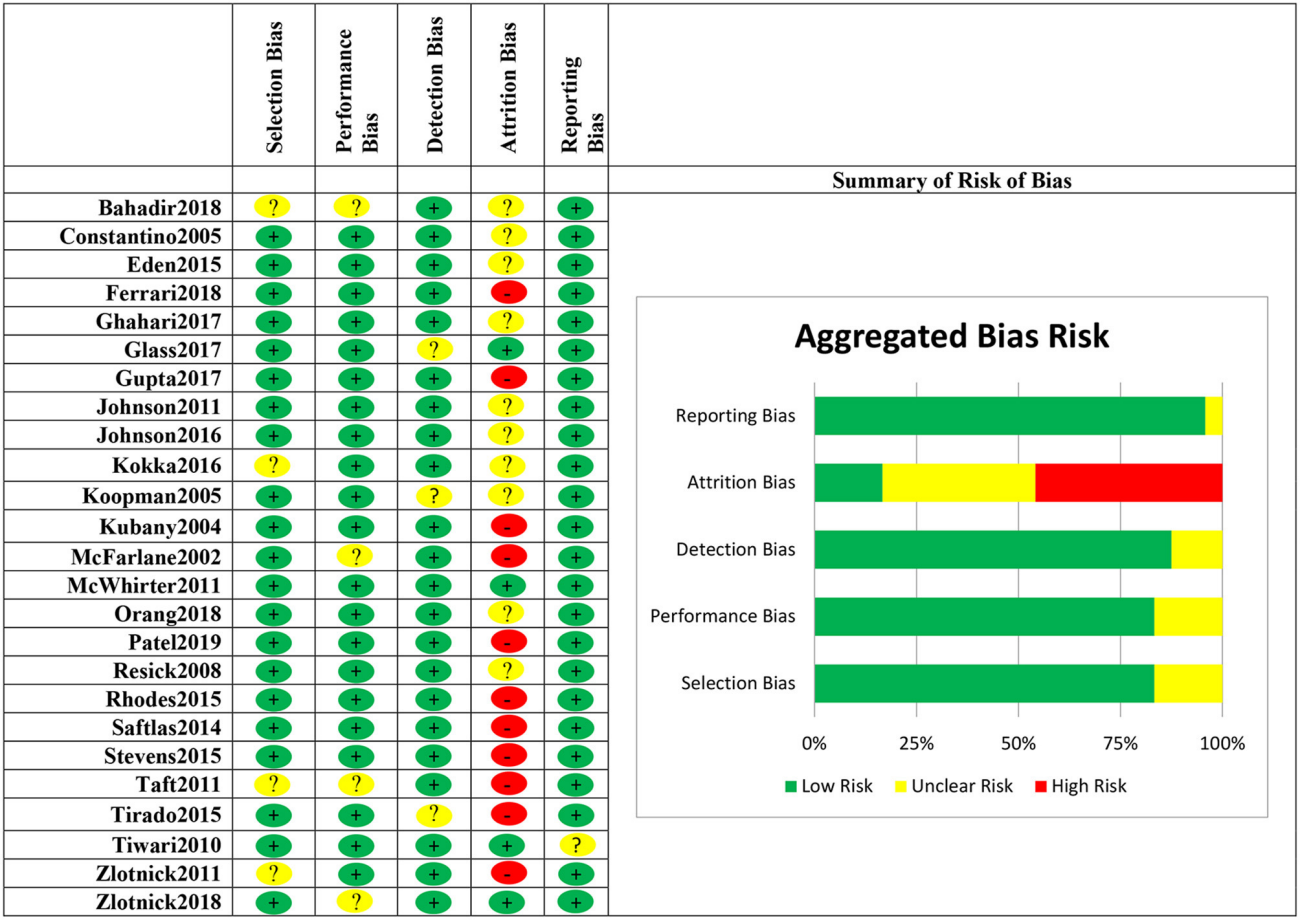
Risk of bias for included studies in the meta-analysis for treatments for female victims of intimate partner violence.

We considered selection bias, performance bias, reporting bias, detection bias, and attrition bias to assess the risk of bias for the current systematic review (Figure 2). Potential sources for the risk of these biases include inadequate randomization into groups, a high dropout rate, and a lack of information about inclusion criteria. Out of the 25 included studies, 15% had a low drop-out rate (<10%), 34% had a moderate drop-out rate (10–19%), and 42% had a high drop-out rate (≥20%). Fifteen percent of studies contained unclear inclusion criteria or high selection and performance bias. This was due to unclear reporting of the study procedures, particularly randomization. Most studies did not contain detection bias since the outcomes were measured consistently across groups, but 12% of studies contained unclear detection bias due to improper or unclear blinding of the outcome assessors. Finally, we found reporting bias to be a low-risk bias for all but one of the studies; this was due to unclear reporting of outcome results.

### Intervention Effects With Minimal Control Groups

#### Mental Health

**Figure 3:**
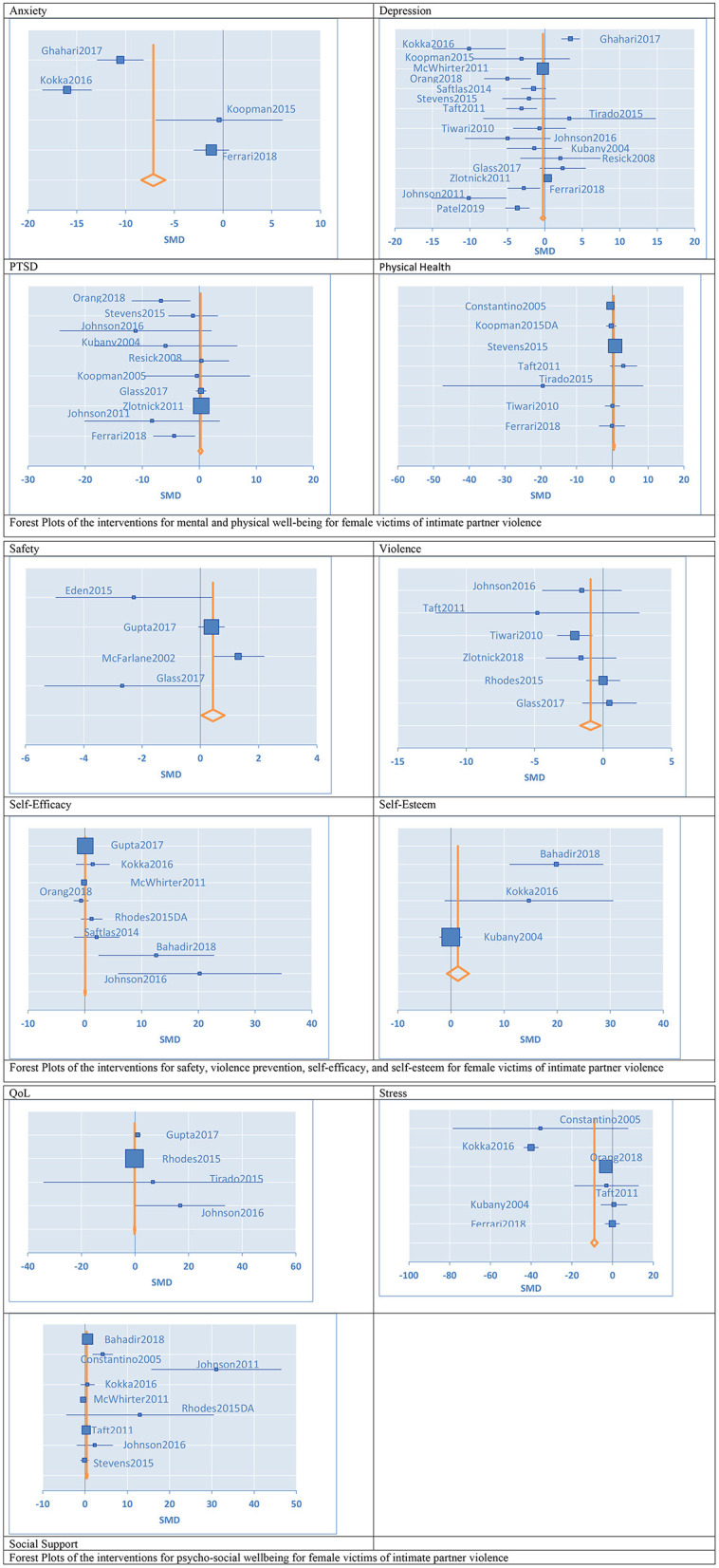
Forest plots of the confidence interval and effect sizes of the interventions for female victims of intimate partner violence.

**Figure 4:**
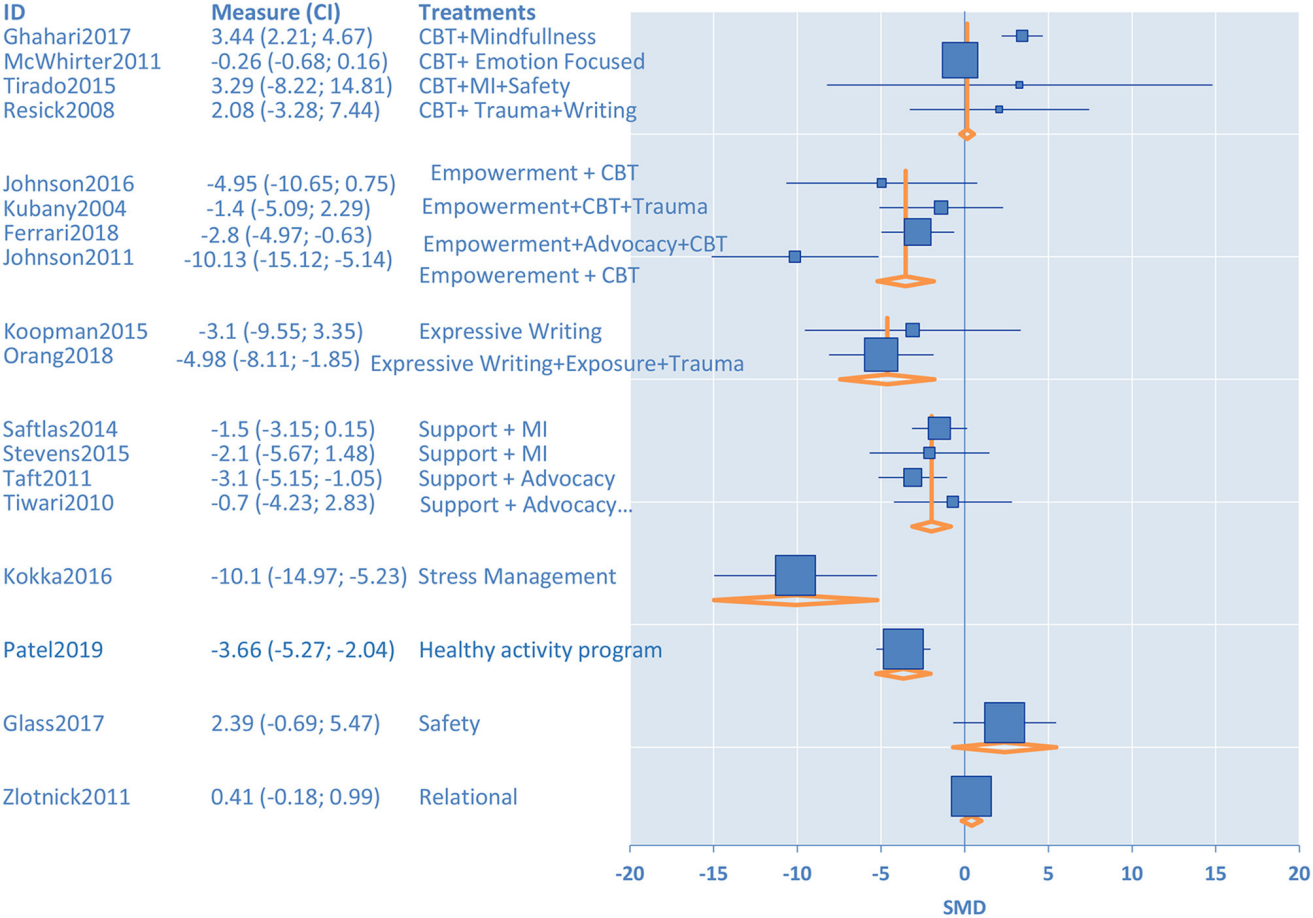
Subgroup analysis for treatment of depression for female victims of intimate partner violence.

**Figure 5:**
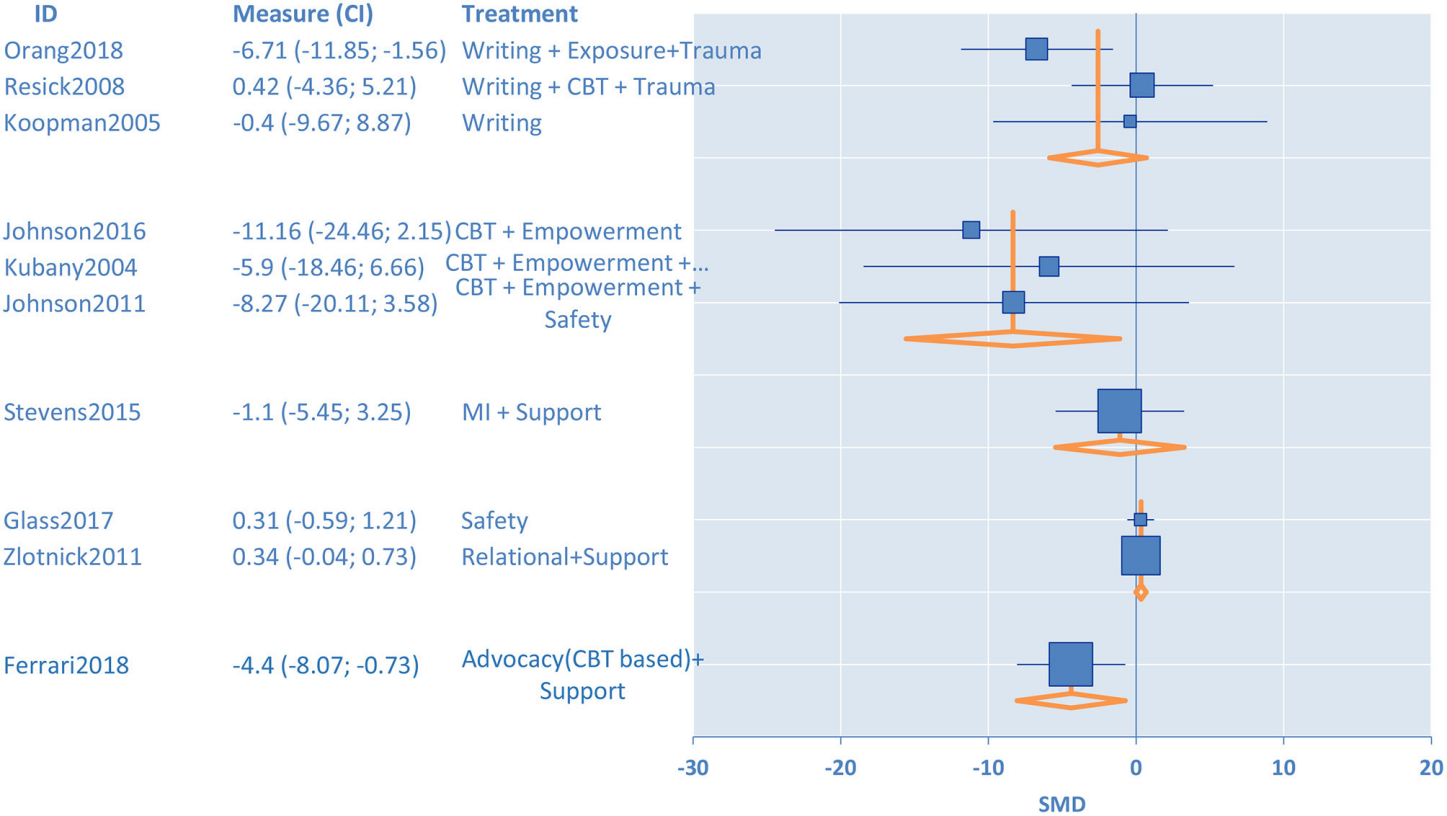
Subgroup analysis for treatment of PTSD for female victims of intimate partner violence.

Four randomized intervention studies with 350 participants measured anxiety as an outcome (Figure 3). Female victims of IPV in the intervention groups had a lower level of *anxiety* at the post-test as compared to participants in the minimal control groups [standardized mean difference (SMD) = −7.15, 95% confidence interval (CI) −8.39 to −5.92] (Table 1). We evaluated eighteen randomized intervention studies with a total of 2,407 female victims of IPV for *depression* as an outcome. Compared to the control groups, participants in treatment groups had lower levels of depressive symptoms at the post-test for the intervention group (SMD = −0.26, CI −0.56 to −0.05) (Table 1). We conducted subgroup analyses to elucidate the differences among intervention programs in terms of their effectiveness in treating depression among IPV victims since there were a sufficiently large number of studies that investigated depression as an outcome (n=18) and these studies had high heterogeneity (*I*^2^ = 86%). The results of the stratified analysis are shown in Figure 4. These results indicated that treatments augmented with expressive writing (SMD = −2.96, CI −5.76 to −0.17), empowerment (SMD = −2.86, CI −4.68 to −1.0), advocacy (SMD = −2.89, CI −4.16 to −0.80), and CBT in combination with empowerment approach (SMD = −3.38, CI −5.36 to −1.4) were the treatments that significantly improved depressive symptoms among female victims. On the other hand, CBT alone (SMD = −1.5, CI −3.15–0.14), programs that address trauma (SMD = −2.36, CI −4.84–0.13), and programs with support components (SMD = 0.80, CI −0.13–1.72) did not show a significant difference in the improvement of depressive symptoms as compared to control groups. Ten intervention studies with a total of 1,454 participants focused on *PTSD* as an outcome. When these studies were pooled together, the PTSD symptoms were not significantly reduced for the participants in the intervention groups compared to control groups (SMD = 0.23, CI −0.12–0.58). We conducted a subgroup analysis to further understand the differences among the intervention programs that considered PTSD as an outcome since these 10 studies had moderate heterogeneity (*I*^2^ = 54%). Our results indicated that treatments utilizing CBT (SMD = −3.07, CI −6.76 to −0.66), and CBT combined with empowerment (SMD = −5.5, CI −9.12 to −1.88) resulted in significantly lower levels of PTSD symptoms as compared to controls. Trauma-focused (SMD = −3.37, CI −7.10–0.35) and expressive writing approaches (SMD = −2.84 CI −6.47–0.79) were not significantly reduced PTSD for the female victims of IPV in the intervention groups compared to control groups through there was a trend in improvement for some participants. These results are shown in Figure 5.

**Table 1:** Results for meta-analysis and subgroup analysis in the meta-analysis for treatments for female victims of intimate partner violence.

**Outcome measures**	**Number of studies**	***n***	**Pooled SMD (CI)**	***I^**2**^ %***	**Test for overall effect**	**Subgroup analysis**	***n***	**SMD [95% CI]**	**Test for subgroup difference**
Anxiety	4, 5, 10, 11	350	−7.15 (−8.39; −5.92)	97	*Z* = −11.39 (*p* < 0.001)^***^	
Depression	4, 5, 6, 8, 9, 10, 11,12, 14, 15, 16, 17,19, 20, 21, 22, 23, 25	2,407	−0.26 (−0.56; −0.05)	86	*Z* = −1.66 (*p* = 0.09)	Support	499	0.80 (−0.13; 1.72)	*Z* = 1.68 (*p* = 0.09)
						Trauma	219	−2.36 (−4.84; 0.13)	*Z* = −1.86 (*p* = 0.06)
						CBT	663	0.52 (−0.35; 1.39)	*Z* = 1.17 (*p* = 0.24)
						Expressive writing	180	−2.96 (−5.76; −0.17)	*Z* = −2.08 (*p* < 0.05)^*^
						Empowerment	674	−2.86 (−4.68; −1.0)	*Z* = −3.07 (*p* < 0.05)^*^
						MI	568	−1.32 (−3.12; 0.47)	*Z* = −1.45 (*p* = 0.15)
						Advocacy	591	−2.48 (−4.16; −0.80)	*Z* = −2.89 (*p* < 0.05)^*^
						CBT + Empowerment	474	−3.38 (−5.36; −1.4)	*Z* = −3.34 (p < 0.001)^**^
Health	2, 4, 11, 20, 21, 22, 23	772	0.39 (0.12; 0.66)	75	*Z* = 2.88 (*p* < 0.05)^*^	Support	786	0.77 (−0.91; 2.45)	*Z* = 0.89 (*p* = 0.37)
						Advocacy	569	0.17 (−1.72; 2.07)	*Z* = 0.18 (*p* = 0.85)
IPV	6, 9, 18, 21, 22, 24	1,482	−0.92 (−1.66; −0.17)	41	*Z* = −2.40 (*p* < 0.01)^**^	
PTSD	4, 6, 8, 9, 11, 12, 15, 17, 20, 25	1,454	0.23 (−0.12; 0.58)	54	*Z* = 1.29 (*p* = 0.19)	Expressive writing	180	−2.84 (−6.47; 0.79)	*Z* = −1.53 (*p* = 0.12)
						CBT	571	−3.7 (−6.76; −0.66)	*Z* = 2.38 (*p* < 0.05)^*^
						Trauma focused	219	−3.37 (−7.10; 0.35)	*Z* = −1.77 (*p* = 0.07)
						CBT + Empowerment	472	−5.5 (−9.12; −1.88)	*Z* = −2.98 (*p* < 0.05)^*^
QOL	7, 8, 18, 22	1,157	−0.16 (−0.36; 0.05)	65	*Z* = −1.49 (*p* = 0.14)	
Safety	3, 6, 7, 13	2,304	0.43 (0.04; 0.83)	77	*Z* = 2.15 (*p* < 0.05)^*^	
Self-efficacy	1, 7, 9, 10, 14, 15, 18, 19	1,552	0.05 (−0.07; 0.18)	64	*Z* = 0.80 (*p* = 0.43)	
Self-esteem	1, 10, 12	204	1.33 (−0.73; 3.39)	91	*Z* = 1.26 (*p* = 0.21)	
Social Support	1, 2, 8, 9, 10, 14, 18, 20, 21	1,006	0.40 (0.20; 0.61)	79	*Z* = 3.84 (*p* < 0.001)^**^	
Stress	2, 4, 10, 12, 15, 21	498	−8.94 (−10.48; −7.40)	99	*Z* = −11.4 (*p* < 0.001)^***^	

*1, Bahadir-Yilmaz and Öz (2018); 2, Constantino et al. (2005); 3, Eden et al. (2015); 4, Ferrari et al. (2018); 5, Liu et al. (2020); 6, Glass et al. (2017); 7, Gupta et al. (2017); 8, Johnson et al. (2011); 9, Johnson et al. (2016); 10, Kokka et al. (2019); 11, Koopman et al. (2005); 12, Kubany et al. (2004); 13, McFarlane et al. (2002); 14, McWhirter (2011); 15, Orang et al. (2018); 16, Patel et al. (2019); 17, Resick et al. (2008); 18, Rhodes et al. (2015); 19, Saftlas et al. (2014); 20, Stevens et al. (2015); 21, Taft et al. (2011); 22, Tirado-Muñoz et al. (2015); 23, Tiwari et al. (2010); 24, Zlotnick et al. (2019); 25, Zlotnick et al. (2011); n, number of participants; I^2^, heterogeneity test; SMD, standardized mean difference; 95% CI, 95% confidence interval; MI, Motivational Interviewing; CBT, Cognitive Behavioral Therapy*.

*** *p < 0.001*,

** *p < 0.01*,

* *p < 0.05*.

#### Violence Reduction and Safety Programs

Six studies with a total of 1,482 participants aimed to diminish violence in the relationship. Overall, interventions that were targeting victims of violence diminished the frequency and severity of the violence victimization at the post-test as compared to the control groups (SMD = −0.92, CI −1.66 to −0.17). Four studies with a total of 2,304 participants aimed to increase safety in the relationship. We found these interventions provide significant improvement in the safety of survivors as compared to control groups (SMD = 0.43, CI 0.04–0.83).

#### Self-Esteem and Efficacy

Three studies with 204 participants specifically measured self-esteem for their interventions. When we pooled these studies together, interventions did not show significant improvement in self-esteem compared to control groups (SMD = 1.33, CI −0.73–3.39). Eight studies with a total of 1,552 participants aimed to improve the self-efficacy of the survivors of IPV. When we pooled these studies together, interventions did not show significant improvement in self-efficacy of the participants as compared to control groups (SMD = 0.05, CI −0.07–0.18).

#### Improving Supportive Environment and Quality of Life

Seven controlled studies with a total of 772 participants investigated a self-reported measure of overall health as an outcome. When pooled together, interventions used in these studies improved the self-reported overall health outcomes at the post-test as compared to the control groups (SMD = 0.39, CI 0.12–0.66). Nine studies with 1,006 participants assessed the impact of IPV interventions on social support. Overall, interventions significantly improved the social support systems for the victims of violence (SMD = 0.40, CI 0.20–0.61). Six studies with 498 participants assessed the impact of IPV interventions on reducing stress for survivors. When pooled together, these studies showed that interventions significantly improved stress levels as compared to control groups (SMD = −8.94, CI −10.48 to −7.40). Four studies reported on QoL with a total of 1,157 participants. Victims' QoL was found to be not affected by the interventions provided (SMD = −0.16, CI −0.36–0.05).

## Discussion

The aim of this study was to explore the effectiveness of psychological interventions for adult women victims of IPV. Although anyone can be a victim of IPV, women are disproportionately affected by it. We identified 25 studies with 4,683 participants. We investigated outcomes including mental health-related problems, self-concept related difficulties as well as supportive treatments. We examined changes in wellbeing by comparing the standardized pre-post-effects in each outcome against the intervention to the control groups. While several prior systematic reviews have explored specific IPV treatment effects on future conflict and violence, we identified several novel findings in this review. First, interventions had more success than control groups in improving anxiety, stress-related issues, improving health and health care utilization, social support, safety, and diminishing further violence. Second, in subgroup analyses, effective interventions for improving depressive symptoms included cognitive-behavioral therapy (CBT) augmented with empowerment, expressive writing, and psychological advocacy programs. Sub-group analyses also indicated that CBT and CBT in combination with empowerment were effective in improving PTSD symptoms.

Many interventions in this study had face-to-face sessions with victims and helped outline safety behaviors, treatment of mental health problems, provided information about community resources, and support victims to improve their wellbeing over the phone or online. Some community-based intervention programs also have telephone support services that were utilized by the victims (Stevens et al., 2015). We also observed that online platforms gained attention for reaching out, empowering victims, and improving safety. These online empowerment programs aim to improve access and utilization of social, justice, and health care services for abused women, which added some personalization to these programs (Glass et al., 2017; Koziol-Mclain et al., 2018).

Among the included studies, we observed that some programs aimed to target a single outcome such as safety (Eden et al., 2015), while others worked from a co-morbidity perspective to address multiple issues that contribute to wellbeing (Kubany et al., 2004; Koopman et al., 2005; Ghahari et al., 2016). Others have added components to improve wellbeing in combination with empowerment (Ferrari et al., 2018) and self-efficacy (Gupta et al., 2017). Local shelters helped with the implementation of these programs (Constantino et al., 2005; Johnson et al., 2011), in a community setting (Eden et al., 2015), and through online platforms (Glass et al., 2017). Community-based intervention programs frequently focused on advocacy, social support, career support, and safety behaviors (McWhirter, 2011; Tiwari et al., 2012; Eden et al., 2015).

### Mental Health

The majority of the studies reported depression and PTSD as their main outcome. Depression can interfere with how victims react to events, normal daily functioning, and how they form relationships with others. Prior research showed that IPV victims are four times more likely to suffer from depression with high rates of suicidality (Anderson et al., 2003). When we pooled all together, the interventions were successful in decreasing depressive symptoms. Detailed subgroup analysis indicated that interventions such as CBT combined with empowerment, empowerment, expressive writing, advocacy programs, and trauma-informed approaches were more likely to lower the depression levels than studies that did not include these interventions.

It can be useful to interpret our findings in the context of a prior meta-analysis conducted on the effectiveness of psychotherapy programs on depression with 53 studies (Cuijpers et al., 2008). The prior metaanalysis concluded that there was no large differential effectiveness among major psychotherapies for the treatment of mild to moderate depression. However, they noted that interpersonal psychotherapy was slightly more effective than others, while supportive treatments were not as effective. Our findings in the context of treating depression in IPV survivors were similar in that supportive treatments were not very helpful for the treatment of depression. However, we found that, for IPV survivors, these supportive treatments can be beneficial for other outcomes such as improving social support and health. Another meta-analysis that investigated the differential effectiveness of mindfulness-based interventions based on 209 studies indicated that such interventions had moderate effectiveness in treating anxiety and depression, while this effect was not stronger than that of traditional CBT (Khoury et al., 2013).

PTSD is another frequently reported mental health problem by IPV victims. Overall, our meta-analysis found that PTSD symptoms were not significantly reduced by the interventions. In contrast, we observed a slight increase in symptoms, which is consistent with the trauma literature in that some treatments may trigger traumatic memories. However, our subgroup analysis indicated that carefully targeted trauma-focused treatments, CBT, and CBT combined with empowerment were promising approaches to improve PTSD symptoms. Expressive writing and relational and safety approaches, on the other hand, did not reach statistical significance. Our finding is consistent with the literature that shows mixed results on the effectiveness of some approaches on improving PTSD symptoms (Abramowitz et al., 2001; Sloan et al., 2015). A similar meta-analysis conducted on the efficacy of psychotherapy treatments for adult childhood sexual abuse survivors with PTSD indicated that trauma-focused treatment was more efficacious as compared to non-trauma-focused treatments (Ehring et al., 2014).

Anxiety is another mental health issue that is more likely to be reported if women have a violent relationship (Mapayi et al., 2012). We found that interventions were successful in lowering the anxiety levels of IPV victims. A prior meta-analysis on the effectiveness of exercise training programs in anxiety reduction with 40 studies found that these programs on stress management resulted in lowering anxiety among adults (Long and Van Stavel, 1995).

### Violence Reduction and Safety Programs

**S**helters, community agencies, NGOs, and government agencies frequently implement safety and violence reduction programs for IPV victims. These programs focused on helping women exit abusive relationships by providing information about safe places, how to seek help, being aware of how to protect themselves before and after a violent altercation. When pooled together, these studies aiming to diminish violence and improve safety in the relationship improved these outcomes.

### Self-Esteem and Efficacy

Past research found that IPV often erodes the victim's self-esteem (i.e., confidence about one's own personal value and worth) and self-efficacy (i.e., confidence in his/her ability to achieve goals). Furthermore, highly correlated issues of emotional abuse such as constant degradation at the hands of abusers can also lead to lower levels of confidence and self-value (Gondolf et al., 2002). Therefore, improving self-efficacy is viewed as a crucial issue by researchers. We observed improvement in self-esteem while there was no improvement observed on self-efficacy when the pre- and post-test data from intervention groups were compared to control groups. It is possible that improving self-efficacy might need a more invested treatment approach, with a longer treatment time.

### Supportive Environment and Quality of Life

Social support, stress, overall self-reported health, and QoL are often interlinked concepts. Survivors of IPV frequently suffer from health problems, excessive stress, difficulty accessing support, and, in turn, have lower QoL. Researchers developed intervention programs to deal with multiple issues that target stress, support, health, and QoL. Studies utilizing seeking help, support, and advocacy approaches reported improved self-reported health outcomes.

### Clinical Implications

The results of this study have few clinical implications. Current interventions have promising results in improving safety and future re-victimization, as well as providing supportive treatments in improving service utilization, social support, and stress management. Methods such as providing a safety plan, psychosocial education materials, and increasing social support were found to help lower the re-victimization rate for women involved in IPV. On the other hand, mental health issues were found to be more complex to treat, particularly depression and PTSD. For depression, CBT combined with empowerment, empowerment, expressive writing, advocacy programs, and trauma work were more likely to lower the depression levels. Targeted trauma-focused treatments, CBT, and CBT combined with the empowerment approach are encouraging methods to improve PTSD symptoms. Overall, if the women feel empowered there is a better chance of improving multiple outcomes such as depression, PSTD, and other underlying issues. Improving confidence in achieving the goals of the victim that is required for the healing process might need more time and more targeted approaches within a safe and supportive environment. Utilizing a complex approach that is taking larger social influences into account can help in improving service utilization for safety, social support, and stress management. Improving the quality of life for women victims of IPV might be more complicated as multiple co-morbidities might be present, hence other basic and essential conditions (i.e., safety, violence reduction), underlying (i.e., depression, anxiety, PTSD), and interwoven issues (i.e., empowerment). These essential, underlying, and interwoven issues need to be identified and treated in combination to have an impact on QoL.

### Limitations and Future Research

Limitations of this study included that many of the studies in the reviewed literature contained minimal controls that consisted of providing IPV literature or giving victims information about community resources. More head-to-head intervention research is needed to understand the comparative effectiveness of these interventions for various outcomes. These comparative effectiveness studies could also be helpful in developing a deeper understanding of mechanisms of change for these interventions. The common problem of high attrition in violence research was observed in this meta-analysis as well. Strategies, such as MI prior to interventions, could be helpful for lowering high drop-out rates in victim populations. Furthermore, future research can also utilize pooled pre-post-test data from the studies without the control condition to better understand these interventions and what works potentially in situations when there are not enough controlled studies in subsequent meta-analyses.

Metanalysis is frequently used in clinical research by integrating the results of a number of independent studies to understand the effectiveness of a course of treatment (Haidich, 2010). This involved assessing the clinical changes in the women's lives as well as the quantifiable statistical effects. In this study, we observed that studies investigating depression, PTSD, and anxiety as an outcome frequently utilized inventories and scales with clinical cut-off scores, indicative of not only statistical but also clinical changes. Furthermore, studies also collected data on qualitative changes while some others did not report this data. For example, Johnson's 2011 study shows that the HOPE intervention was successful in reducing risk of re-abuse in women. They also reported higher levels of empowerment and social support months after the intervention was over. Kubany's study showed that 70% of the women had good end-state functioning, the reduction of PTSD and depression symptoms, 6 months after the study. In future research, more information on the qualitative experiences of the women going through these changes will be instrumental.

In summary, IPV creates negative physical and psychological effects on the wellbeing of victims and their families. The effects of IPV are long-lasting and require treatment focusing on the complexity of the person by improving mental health issues, safety, and support. Overall, if women feel empowered, the chances of improving multiple outcomes are higher including depression and PSTD.

## Data Availability Statement

The raw data supporting the conclusions of this article will be made available by the authors, without undue reservation.

## Author Contributions

GK, EK, and PK contributed to data analysis, writing, and editing. GK contributed to conceiving the idea, facilitated data collection, and management. EK, NJ, and PK contributed to reviewing the articles. PK and NJ contributed to data collection. SB contributed to supervising the data analysis and meta-analysis process, reading, reviewing, and editing. All authors contributed to the article and approved the submitted version.

## Funding

This publication was made possible by R01-LM012518 from the National Library of Medicine.

## Author Disclaimer

The contents are solely the responsibility of the authors and do not necessarily represent the official views of the NIH.

## Conflict of Interest

The authors declare that the research was conducted in the absence of any commercial or financial relationships that could be construed as a potential conflict of interest.

## Publisher's Note

All claims expressed in this article are solely those of the authors and do not necessarily represent those of their affiliated organizations, or those of the publisher, the editors and the reviewers. Any product that may be evaluated in this article, or claim that may be made by its manufacturer, is not guaranteed or endorsed by the publisher.
