# Supplementary material for: Treatments for Female Victims of Intimate Partner Violence: Systematic Review and Meta-Analysis
Source: Front Psychol. 2022 Feb 4;13:793021. doi: 10.3389/fpsyg.2022.793021 (PMC8855937; doi:10.3389/fpsyg.2022.793021)
Supplement: Supplementary file 1 [file Table_1.docx]

| Supplementary Table 1.  *Characteristics of included studies in the meta-analysis for treatments for female victims of intimate partner violence* | | | | | | |
| --- | --- | --- | --- | --- | --- | --- |
| **Study** | **Sample** | **Sample Characteristics**  **#, Age (SD), Race** | **Treatment**  **Arms** | **Intervention** | **Results/Attrition Rate** | **Outcomes** |
| Bahadir2019 | Women at the Family Counseling Center (FCC) | Intervention:N=30, Mean age= 40.7  Control: N=30, Mean=34.6 | Empowerment program intervention    Control:  Non-empowerment focused support | Developing self-awareness, increasing self-esteem, decreasing learned helplessness, increasing learned resourcefulness, making sense of violence, managing violence, increasing effective coping, using social resources to cope with violence | Women in the intervention group showed significant improvements in their Self Esteem Inventory, improved their learned resourcefulness, increased self-confidence  Drop-out rate is not inferred in the article | Self Esteem, Learned resourcefulness, Self-confident approach, Helpless approach (decreased), Yielding approach, Optimistic approach, Social support seeking approach |
| Constantino2005 | Individuals arrested past year for substance abuse issues and seeking treatment | N= 24, Mean age = 35.45 (7.25)  70.8% White | Social Support Intervention + SSS    No Treatment Control (Free-flowing chat session) + SSS | The intervention was designed to provide information to women about resources, create an environment to chat with the counselor, and gave them time to access resources when available  Focused on 4 different functions of social support: Belonging, Evaluation, Self-Esteem, Tangible Support (BEST) | Participants in the SSI group had greater improvement in psychological distress symptoms and social support  82% completed the treatment, Drop out rate: **18%** | Social Support, Stress |
| Eden2015 | Women that experienced physical, sexual, emotional abuse in the general community | N=826, Mean age = 33.34 (10.63)  63.4% White | Safety Decision Aid  Control: Usual safety planning resources | The intervention included 3 components: Safety priority-setting activity (personalized), danger assessment, and safety planning strategies and resource information based on response to the first two components  Control: Received resources but no feedback and no safety-priority setting | Participants in the intervention group had a greater reduction in total decisional conflict at post-test.  Drop out rate inferred as **14.3%,** | Safety |
| Ferrari2018 | Women seeking help from DVA agencies | N=249  Mean age: Intervention: 33 (11)  Control:  34 (10) | Specialist Psychological Advocacy (SPA)  Control: Usual Care (Support + Advocacy) | The intervention included receiving eight SPA sessions alternated with regular advocacy sessions. SPA sessions included cognitive-behavioral psychological techniques. | Participants in the intervention group had a greater reduction in psychological distress and depression  Drop out rate inferred to be **36%** | PTSD, Depression, Anxiety, IPV |
| Glass2017 | Currently abused Spanish or English speaking women | N=725 Mean age: 33.41 (10.64)  63.67% White | Internet Safety Decision Aid  Control: Regular safety information online | The intervention included priority setting activities, risk assessment, tailored feedback, and safety plans | There were no significant differences in IPV, depression, or PTSD between both groups  Drop out rate was between **8.5%-9.2%** | Safety, Depression, PTSD |
| Ghahari2017 | Women victims of domestic violence in Tehran | N=30  Majority of the women age ranging from 20 to 25 | Mindfulness-based cognitive therapy  VS  Control: Waiting list until the end of the intervention | Treatment consisted of 8 sessions (45 min)  Sessions covered topics such as meditation, training consciousness, explaining emotions, training mindfulness in relationships, | Results showed that mindful CBT can affect reducing depression and state-trait anxiety in women victims of violence  Drop out rate inferred as **23%** | Depression, Anxiety |
| Gupta2017 | Women experiencing recent IPV through screening within public health clinics in Mexico City | N=950 (30.12,7.28)  Control: (29.6, 7.03) | Nurse-delivered intervention  Control: Screening/referral card from nurses | The intervention included integrated IPV and health screening assessment, supportive care, safety planning, harm reduction counseling, and assisted referrals.  The intervention was delivered by trained nurses. | There were no significant differences in safety planning, use of community resources, or mental quality of life between the treatment and control. The intervention involved empowerment, cognitive-restructuring, and establishing long term support.  conditions.  Drop out rate inferred as **30.7%** | QoL, Safety, Self-Efficacy, IPV |
| Johnson2011 | Residents of an inner-city battered women’s shelter that had experienced IPV | N=70 (32.55,  African American: 35%  White: 30% | Intervention: HOPE (Helping overcome PTSD through Empowerment) +SSS  CBT therapy  Control: Standard Shelter Services (SSS) | HOPE intervention included three stages: establishing safety, remembrance and mourning, and reconnection. Standard shelter services included case management, a supportive environment, and attendance of education groups offered through the shelter. | Results suggested that patients in the intervention group reported fewer depression symptoms over follow-up, but had no change in PTSD diagnosis  Drop out rate inferred as **7.9%** | PTSD, Depression |
| Johnson2016 | Residents of battered women’s shelters | N=60 Mean age: 33 (10.48)  African American: 56.7%  White: 43.3% | Helping to overcome PTSD through Empowerment(HOPE) + SSS    VS  SSS (Standard shelter services) | The HOPE intervention is cognitive-behavioral therapy and empowerment-based individual treatment that includes elements of cognitive restructuring and skill-building | Women in the HOPE intervention group reported less severe PTSD and depression symptoms and were more likely to report being employed at the follow-up  Drop out rate is inferred to be **20%** | PTSD, Depression |
| Kokka2016 | Greek women who are (or have) experienced IPV, voluntary participation | N=60  Intervention: N=30 Mean age: 47.23 (9.96)  Control: N=30 Mean age: 48.77 (8.90) | Stress Management Program  Control: Informed about stress and healthy lifestyle | The intervention included informative lectures on stress, guided breathing, PMR, dietary counseling, relaxation techniques | There was a significant reduction in depressive symptoms and perceived stress levels in the intervention group. There was also an increase in social support.  No drop out rate. | Depression, Stress, Social Support |
| Koopman2005 | Women in the San Fransisco area that are victims of IPV | N=59,  Mean age= 36.5 (SD=8.9),  68% Caucasian | Intervention: Expressive Writing  Control: Neutral writing | The expressive writing intervention included writing about the most traumatic experience of the victim’s life  The neutral writing task, women were asked about how they used their time | Results indicated that women who were more depressed at baseline demonstrated a significantly greater decrease in depression when assigned to the stressful writing group  Drop out rate inferred as **20%** | Depression, PTSD, Pain |
| Kubany2004 | Formerly battered women referred to victim service agencies in Hawaii | N=125  Mean age: 42.2 (10.1)  52% White | Cognitive Trauma Therapy for Battered Women with PTSD  Control: Delayed CTT-BW | The intervention contained treatment elements from CBT such as psychoeducation about PTSD and stress management. It also included modules that focus on self-advocacy and assertive communication skill-building | Results indicated that women in the immediate intervention no longer met diagnostic criteria for PTSD at post-therapy assessment. Depressive symptoms were also lowered  Drop out rate inferred to be **26.9%** | Depression, PTSD |
| McFarlane2002 | Female applicants at a DA’s office that qualified for a protection order against a sexual intimate | N= 150,  Intervention = 75Mean age = 30.25 (7.87)  40% White  VS  Control: N= 75, Mean age = 34.61 (9.91)  72% White | Safety Intervention Protocol  Control: Standard Services | The intervention consisted of standard services of the district attorney’s office + six safety intervention telephone calls | Participants in the intervention group showed significant adoption of safety behaviors  Drop out rate inferred as **25%** | Safety |
| McWhirter2011 | Women residing in temporary family homeless shelters | N=46, Mean age= 30  47% White | Emotion-Focused  VS  Goal-Oriented | The emotion-focused intervention involved behavioral and gestalt therapeutic interventions  VS  The goal-oriented intervention drew on the CBT approach with motivational interviewing and the transtheoretical model | Women in both treatment groups reported decreases in depression and an increase in self-efficacy  There was a greater increase in social support among emotion-focused participants  Drop out rate inferred as **8%** | Depression, Social Support, Self-Efficacy |
| Orang2018 | Women in Tehran that experienced IPV in the last year with a PTSD diagnosis | N=45  NET: N=24 Mean age: 38.04 (9.69)  (TAU): N=21 Mean age: 37.28 (7.92) | Net Exposure Therapy  VS  Control: Treatment as Usual (TAU) | NET: Intervention involved patient speaking about their traumatic life experience to shape into a written narrative of their life  TAU: Intervention included commonly used psychotherapy for abused women in Iran including life skill training and supportive counseling | NET intervention participants showed a greater reduction in PTSD, depression symptoms, and perceived stress than the TAU group  Drop out rate inferred from the treatment as **22%** | PTSD, Depression, Stress |
| Patel2019 | Married women in Goa that experienced IPV | N=242  Mean age: Unknown | Healthy Activity Progam (HAP)  Vs  Control: Enhanced Usual Care (EUC) | HAP: Intervention included eight sessions that contained psychoeducation on activity, mood, behavior monitoring, etc  EUC: Participants were given usual care, but providers were given more information about psychological status | Participants that had higher activation (HAP) also had lower depressive symptoms and higher social support  Drop out rate inferred to be **29.41%** | Depression, IPV, Social Support |
| Resick2008 | Women from the St.Louis area that experienced IPV | N=150 Mean age: 35.4 (12.4)  62% White | Cognitive Processing Therapy (CPT) + Written Accounts (WA)  Control: None | The CPT intervention included education about PTSD, assignments to write about traumatic events, Socratic questions, and teaching clients to challenge their beliefs | Participants improved with a reduction of PTSD symptoms, depression, and anxiety  Drop out rate is **15%** | PTSD, Depression |
| Rhodes2015 | Female patients that were affiliated with 2 urban academic EDs in Philadelphia | N=592 Mean age: 32 (5.0)  78.5% African American | Motivational Intervention  VS  Assessed Control  VS  Control (no-contact) | The intervention focused on brief ED interventions targeting drinking and used empowerment theoretical frameworks, followed by a telephone booster at 10 days | Results showed improvement of social support for the intervention group, but no change in IPV or drinking between the intervention and control groups  Drop out rate inferred from the treatment as **29%** | QoL, Social Support, IPV |
| Saftlas2014 | Women that had experienced IPV in the past year from a family planning clinic | N= 306 Mean age:  84% White | Motivational Interviewing  Control: Community-based resources | The intervention involved identifying steps that women could take to improve social support, QoL, and emotional health | There was an increase in self-efficacy and a decrease in depressive symptoms among intervention women. Self-efficacy scores were similar between both groups.  Drop out rate inferred from the study as **33.4%** | QoL, Self-Efficacy |
| Stevens2015 | Women from a Midwestern pediatric ED that reported IPV within the past year | N=253  Intervention: Mean age: 28.8 (8.3)  Control: Mean age: 29.5 (8.5)  46.5% White | Telephone Support Services (TSS)  VS  EUC (Control) | The intervention involved five phases that included educating women about community resources, identifying appropriate agencies, and follow-up calls. The interventionist relied heavily on motivational interviewing (respecting priorities of the woman, using reflective listening) | There was a lack of intervention effect for TSS. The TSS and EUC groups do not differ on any outcome variable  Drop out rate inferred as **25-30%** | Depression, PTSD, IPV, Social Support |
| Taft2011 | Women in Australia attending GPs or MCH nurses that were pregnant or had a child under five | N=133 Intervention: Mean age: 32 (6.7)  Control: Mean age: 32.4 (5.8) | Mentor Intervention  Control: Regular clinician care | The intervention included 12 months of weekly home visits from trained and supervised local mothers that offered advocacy, referrals, befriending, and parent support | There was weak evidence for suggesting that the intervention arm reduced depression, but there was reduced partner violence with mentored women  Drop out rate inferred to be **45%** | Depression, Social Support, IPV |
| Tirado2015 | Women from two outpatient drug treatment centers in Barcelona that reported IPV | N=14 Mean age: 40 (8.81) | Intimate Partner Violence Therapy-Cognitive Behavioral Therapy  Control: Treatment as usual at drug treatment center | The intervention consisted of enhancing motivation for wellness, identifying triggers for drug use, dealing with IPV, identifying PTSD triggers, and building relationship safety | Results showed no difference in the reduction of depressive symptoms or an increase in QoL between both groups, but overall participants in both groups showed similar results.  Drop out rate inferred to be **28.5%** | QoL, Depression, Substance Use |
| Tiwari2010 | Community-dwelling Chınese women that screened for IPV | N=200  Intervention: Mean age: 38.18 (7.61)  Control: Mean age: 37.99 (9.07)  100% Asian | Advocacy Intervention  Control: Community services | The intervention consisted of two components: empowerment that included protection and enhanced choice-making, as well as telephone social support | The results did not show a clinically meaningful improvement in depressive symptoms  No dropouts | Depression, IPV |
| Zlotnick2018 | Perinatal women seeking mental health treatment | N=53 Mean age: 28 (5.3)  Intervention:  White 28.5% Hispanic, 60.7%  Control:  White 40%  Hispanic 36% | Strength for U in Relationship Empowerment (SURE)  Control: Watching segments of popular television shows | The intervention included a computerized intervention that has aspects of MI by increasing participants awareness and education | Results showed that IPV decreased significantly, and there was a reduction in emotional abuse as well  No dropouts. | IPV |
| Zlotnick2011 | Low-income pregnant women with recent IPV | N=54 Mean age: 23.8 (4.6)  Hispanic = 42.6%  White = 38.9% | Interpersonal Psychotherapy  Control: Community resources | The intervention included strengthening social relationships and placing an emphasis on the enhancement of social support | There was no difference for the intervention group in terms of major depressive episodes and didn’t reduce the risk of PTSD long term  Drop out rate was inferred at **34%** | PTSD |
